# The emerging roles of protein arginine methyltransferases in antiviral innate immune signaling pathways

**DOI:** 10.3389/fmicb.2023.1322929

**Published:** 2023-12-05

**Authors:** Hui Nie, Qingchao Li, Wei Pan

**Affiliations:** Key Laboratory of Animal Resistance Biology of Shandong Province, College of Life Sciences, Shandong Normal University, Jinan, China

**Keywords:** protein arginine methyltransferases, post-translational modification, innate immunity, arginine methylation, viral infection

## Abstract

The Protein Arginine Methyltransferases (PRMTs) family is involved in various biological processes, including gene transcription, pre-mRNA splicing, mRNA translation, and protein stability. Recently, mounting evidence has shown that PRMTs also play critical roles in regulating the host antiviral immune response, either in an enzymatic activity dependent or independent manner. This review aims to provide an overview of the recent findings regarding the function and regulatory mechanisms of PRMTs in the antiviral response. These findings have the potential to aid in the discovery and design of novel therapeutic strategies for viral infections.

## Introduction

**Figure 1:**
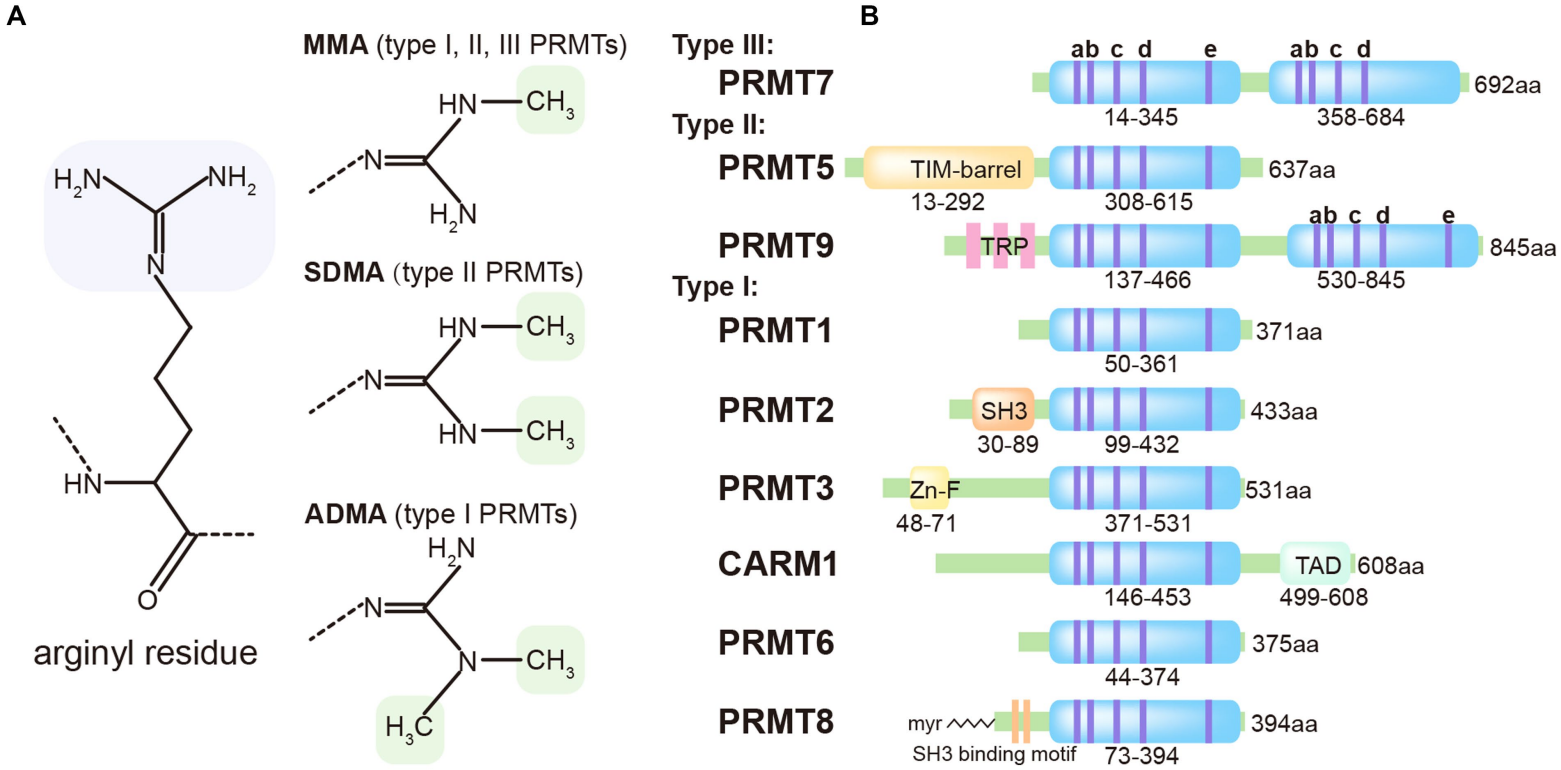
Classification of methyl-arginines and PRMTs based on their types and structures. **(A)** Three distinct types of PRMTs catalyze the methylation of arginyl residues in specific proteins. **(B)** Nine PRMTs were presented with catalytic core (light blue) and unique signatures (bluish violet lines) with high sequence similarity (a, Motif I: VLD/EVGXGXG; b, Post-I: V/IXG/AXD/E; c, Motif II: F/I/VDI/L/K; d, Motif III: LR/KXXG; e, THW loop).

The protein Arginine Methyltransferases (PRMTs) enzyme family consists of 9 members that transfer the methyl group to the arginine residue. Based on their product specificity, PRMTs can be classified into three categories: Type I, which includes PRMT1, PRMT2, PRMT3, PRMT4, PRMT6, and PRMT8; Type II, which includes PRMT5 and PRMT 9; and Type III, which includes only PRMT7 (Blanc and Richard, 2017). Both Type I and Type II PRMTs catalyze the formation of monomethylarginine (MMA). However, Type I PRMTs can introduce a second symmetrical methyl group on the guanidino group of the arginine residue, while Type II PRMTs introduces an asymmetrically methyl group. Type III PRMTs exclusively produces only MMA (Figure 1A).

PRMTs primarily function by methylating arginine residues of target proteins. This modification does not affect the charge state or the capacity to form electrostatic interactions of arginine residues, but it does increase their size and hydrophobicity while reducing their ability to form hydrogen bonds (Tewary et al., 2019; Al-Hamashi et al., 2020). Consequently, arginine methylation has a significant impact on protein-DNA/RNA and protein–protein interactions, thereby modulating various biological pathways (Xu and Richard, 2021). For instance, PRMTs can methylate arginine residues in histones to regulate chromatin restructure. Methylated histones can either activate or repress transcription, depending on the specific marks present on the histones (Di Lorenzo and Bedford, 2011). In addition to histones, PRMTs also methylate various non-histone proteins that play important functional roles, such as innate sensors and downstream adaptors. Therefore, PRMTs have the ability to modulate viral replication by regulating the antiviral response.

### The critical antiviral innate immune responses pathways

Innate immunity serves as the first line of defense against viral infections, protecting the host. This defense is initiated by pathogen-recognition receptors (PRRs) that detect the pathogen-associated molecular patterns (PAMPs) of viruses. Subsequently, innate immune signaling pathways are activated, leading to the induction of type I interferon (IFN-I) expression. IFN-I, in turn, binds to IFN-I receptors, triggering the expression of antiviral factors that inhibit viral infections. Based on the location and recognized PAMPs of PRRs, antiviral signals can be categorized into Toll-like receptor (TLR) signaling pathways, RIG-I-like receptor (RLR) signaling pathways, and DNA sensing signaling pathways.

### TLRs signaling pathway

Toll-like receptors (TLRs) are essential class of PRRs which mainly located in plasma membrane and endosome of antigen presenting cells (Kawai and Akira, 2007). TLRs contains 10 members in humans, among them TLR3, TLR4, TLR7, TLR8, and TLR9 are involving in triggering the expression of IFN-a, β, and γ, which play critical roles in antiviral immunity. After recognizing PAMPs, TLRs activates NF-κB through MyD88-dependent or independent pathway. In the MyD88-dependent pathway, the interaction between TLRs and MyD88 promote the recruitment of TRAF6 and activation of MAPK and IKK complex. This classical pathway causes the expression of pro-inflammatory cytokines. The MyD88-independent pathway was activated by recruiting TRIF to TLR3 and TLR4, which subsequently bind with TBK1 and IKKi to mediated the phosphorylation of IRF3, eventually driving the expression of type I IFN (Zhang et al., 2007).

### RLRs signaling pathway

The RIG-I-like receptors (RLRs), which include RIG-I, MDA5, and LGP2, play a pivotal role in detecting viral RNA in the cytoplasm, initiating an antiviral immune response. When these receptors detect viral single or double-stranded RNA, the structure of RIG-I and MDA5 undergoes a conformational change, resulting in the release of the CARD domains, which then associate with MAVS. This interaction triggers the formation of MAVS filaments and recruits and activates TBK1. Activated TBK1 directly binds to IRF3, IRF7, and IκBα, promoting their phosphorylation, ultimately leading to the formation of the enhanceosome. The enhanceosome binds to the promoter of IFN-I, initiating its expression. Unlike RIG-I and MDA5, LGP2 lacks CARD-like domains and can only competitively bind to pathogen-associated molecular patterns (PAMPs). As a result, LGP2 is considered a negative regulator in the RLR signaling pathway (Satoh et al., 2010; Loo and Gale, 2011).

### DNA sensing signaling pathway

Recently, numerous sensors have been characterized for their ability to detect viral DNA, which possesses unique molecular features distinct from host DNA. Among these pattern recognition receptors (PRRs), cyclic GMP-AMP synthase (cGAS) has emerged as the dominant cytosolic DNA receptor during DNA virus or retrovirus infections (Sun et al., 2013). Upon binding with DNA, cGAS is activated, leading to the synthesis of cyclic GMP-AMP (cGAMP). This second messenger, cGAMP, then binds to and activates Stimulator of Interferon Genes (STING) at the endoplasmic reticulum. The activation of STING results in the formation of tetramers, and it translocates from the endoplasmic reticulum to endoplasmic reticulum-Golgi intermediate compartments. Subsequently, STING recruits TBK1 and interferon regulatory factor 3 (IRF3), ultimately leading to the production of type I interferon (IFN-I) (Wu et al., 2013).

### IFN-I signaling pathway

Interferon-I (IFN-I), induced by the pathways described above, exerts antiviral functions by binding to the IFNAR1/2 receptors, recruiting and activating the Tyk2 and Jak1 kinases. Subsequently, the activation of Tyk2 and Jak1 promotes the phosphorylation of STAT1 and STAT2, leading to their heterodimerization. This heterodimer interacts with IRF9, forming the ISGF3 complex, thereby inducing the expression of interferon-stimulated genes (ISGs). These ISGs act to inhibit viral propagation at various stages of the viral life cycle through a variety of mechanisms (Ivashkiv and Donlin, 2014).

### The roles of PRMTs in regulating innate immune responses

**Figure 2:**
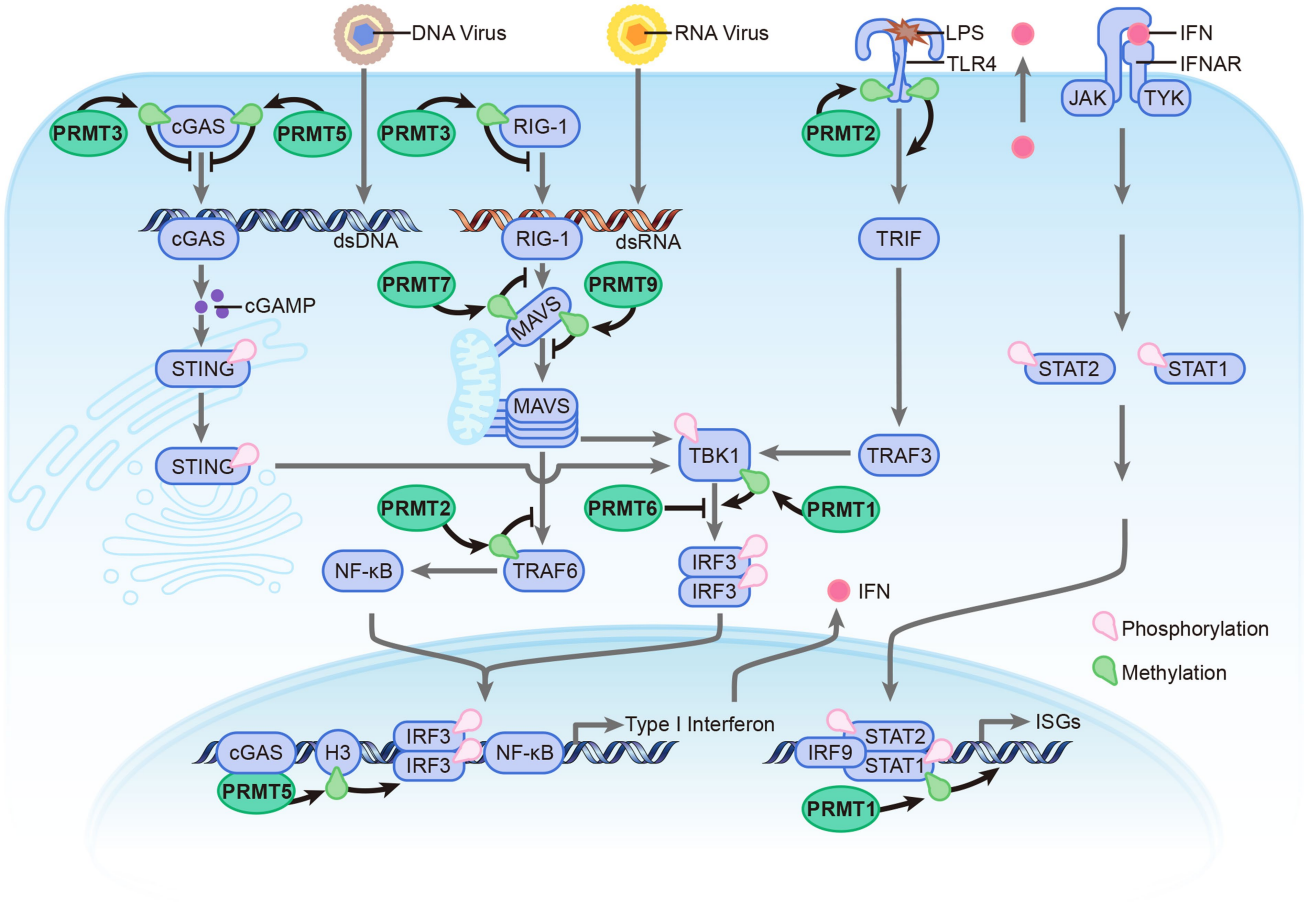
Overview the roles of PRMTs in antiviral immune response. The PRMTs family include: PRMT1, PRMT2, PRMT3, PRMT5, PRMT6, PRMT7, and PRMT9 involved in regulating antiviral immune response induced by RNA, DNA, and LPS. PRMTs modulate antiviral immune response mainly via methylation of innate sensors and downstream adaptors. Compared with other PRMTs, PRMT6 could directly bind with those proteins to hinder the Innate Immune signaling in a enzymatic activity independent manner.

Recent evidence suggests that post-translational modifications (PTMs) play a vital role in regulating PRR-dependent immune responses by targeting innate sensors and downstream adaptors (Kumar et al., 2020). Arginine methylation, mediated by PRMTs, is involved in regulating antiviral immune response by modulating the methylation of these proteins (Figure 2). This paper discusses various arginine methyltransferases that play a crucial role in regulating innate immunity and elucidates their mechanisms in modulating the antiviral response.

### PRMT1

PRMT1 is expressed ubiquitously in most tissues and accounts for up to 85% of all PRMT activity in cultured RAT1 cells and mouse liver tissue (Tang et al., 2000). It is widely recognized as the primary enzyme responsible for monomethylation and asymmetric dimethylation of Arg-3 on histone H4, which is required for the activation of nuclear hormone receptors (Strahl et al., 2001).

Beyond its role in modulating chromatin structure and function, PRMT1 has also been demonstrated to regulate the antiviral immune response. Upon infection with SeV or HSV-1, PRMT1 forms oligomers possessing methyltransferase activity. In the subsequent process, TBK1 is recruited to these PRMT1 oligomers where it is subjected to asymmetric methylation at R54, R134, and R228. This methylation process facilitates TBK1 aggregation and triggers its trans-autophosphorylation, which is essential for its activation. Once activated, TBK1 directly phosphorylates IFN regulatory factors 3 and 7 (IRF3 and IRF7), encouraging their dimerization and nuclear translocation, which in turn stimulates the production of IFNs. When PRMT1 is knocked down, the expression of IFN -β post infection with SeV and HSV-1 is notably attenuated. This simultaneous promotes the replication of these viruses, making mice with PRMT1 knocked out more prone to DNA and RNA virus infections compared with their WT counterparts (Yan et al., 2021). Furthermore, besides its important role in regulating the expression of IFN, PRMT1 also influences the downstream signaling pathway of IFN. Via methylating STAT1 at Arg-31, PRMT1 enhances the transcriptional activation of STAT1 (Mowen et al., 2001). During Hepatitis B virus infection, it was observed that IFN-α signaling can be inhibited due to an upregulation of Protein Phosphatase 2A (PP2A). PP2A, in turn, inhibits PRMT1, the enzyme responsible for catalyzing the methylation of STAT1, a prominent transducer of the IFN-α signal. The end result is a hypomethylated STAT1, whose activity is reduced because it becomes bound by its inhibitor, PIAS1 (Christen et al., 2007).

### PRMT2

PRMT2, also known as HRMT1L1, is primarily localized in the nucleus. Compared to other PRMTs, PRMT2 exhibits weak methyltransferase activity, making it challenging to investigate its role in cellular processes. Several reports suggest that PRMT2 interacts with regulatory proteins and forms oligomers with PRMT1 (Cura and Cavarelli, 2021). Recent studies have shown that PRMT2 also plays a role in modulating the antiviral immune response. The overexpression of PRMT2 inhibits RLR signaling while also suppressing the expression of type I IFNs and IFN-stimulated genes, which, in turn, enhance viral replication. In stark contrast, the knockout of PRMT2 induces opposing effects. PRMT2 functions as a negative regulator of RLR signaling, through mechanisms that are both dependent and independent of its enzymatic activity. For one, PRMT2 catalyzes the asymmetric dimethylation of arginine on TRAF6 at position 100, thereby inhibiting TRAF6’s autoubiquitination. This inhibition prevents TRAF6 from forming K63-linked polyubiquitin chains, thus leading to the inactivation of the NEMO-TANK-TBK1/IKKε complex. Simultaneously, the N-terminus of PRMT2 competes with MAVS for the binding site on TRAF6. This competition blocks the recruitment of TBK1/IKKε to MAVS by TRAF6. Both of these pathways culminate in the inhibited phosphorylation of IRF3/7, which subsequently results in the downregulating of type I IFNs (Zhu et al., 2021b). Additionally, PRMT2 interacts with the cytoplasmic domain of TLR4, leading to the methylation of R731 and R812 on TLR4. Further studies have demonstrated that the methylation of R812 is necessary for the recruitment of IRF3 by TLR4, thereby promoting the expression of IFN-β (Wang et al., 2021).

### PRMT3

PRMT3 is expressed ubiquitously in the cytoplasm. It consists of a C-terminus catalytic domain and an N-terminus zinc-finger domain (Figure 1B), which are involved in recognizing RNA-associated substrates and regulating the catalytic activity of enzyme by interacting with different partners (Frankel and Clarke, 2000). Acting as a negative regulator of cytosolic RNA and DNA sensors, PRMT3 interacts with RIG-I, MDA5, and cGAS, promoting asymmetric dimethylation at positions R730 on RIG-I, R822 on MDA5, and R111 on cGAS. These modifications impede the RNA-binding abilities of RIG-I and MDA5 and diminish the DNA-binding capacity and oligomerization of cGAS. Together, these alterations inhibit downstream type I interferon production. Further research has shown that mice with a single copy of Prmt3 deleted or those treated *in vivo* with a PRMT3 inhibitor exhibit enhanced resistance to RNA and DNA viral infections (Zhu et al., 2020; Zhu et al., 2023).

### PRMT5

As an important type II arginine methyltransferase, PRMT5 functions by interacting with MEP50 to form a unique hetero-octameric complex (Motolani et al., 2021). PRMT5 is involved in various biological processes, particularly in immunity-related processes. It promotes the differentiation of T-helper 17 (TH17) cells and regulates the function of B cells (Litzler et al., 2019; Webb et al., 2020). Additionally, PRMT5 plays a role in modulating the antiviral innate immune response. As a direct binding partner of cGAS, PRMT5 facilitates the symmetrical dimethylation of the Arg^124^ residue within cGAS. Subsequent research has indicated that such methylation by PRMT5 weakens the antiviral response mediated by cGAS, chiefly by impeding its ability to bind DNA. Moreover, the oral delivery of PRMT5 inhibitors substantially enhances the resilience of mice against HSV-1 (Herpes Simplex Virus-1) infections and prolongs their survival time post-infection (Ma et al., 2021). PRMT5 also participates in regulating the RLR signaling pathway by catalyzing the symmetric dimethylation of histone H3 arginine 2 at the IFN-β and IFN-α4 promoters. This modification promotes the binding of IRF3 to these promoters. Furthermore, the catalytic activity of PRMT5 relies on its interaction with cGAS (Cui et al., 2020).

### PRMT6

PRMT6, which abundantly located in the nucleus, acts as a transcriptional repressor by automethylating its R35 residue. As a protein induced by viral infection, PRMT6 has been identified as a negative regulator of antiviral innate immunity. Stable overexpression of PRMT6 in cells leads to a marked reduction in IFN-I mRNA levels following infection with VSV. In contrast, a deficiency of PRMT6 bolsters the antiviral immune response by promoting IRF3 activation and boosting the production of type-I interferon. Instead of methylating the innate immune sensors and their associated adaptors, PRMT6 inhibits the antiviral response by directly binding to IRF3, thereby acting as an impediment to the TBK1–IRF3 signaling axis to inhibit the activation of IRF3 (Jiang et al., 2019; Zhang et al., 2019; Jiang et al., 2022).

### PRMT7

PRMT7 is a unique type III PRMT that catalyzes the formation of monomethylation on its substrates. PRMT7 is involved in various cellular processes, including DNA damage response, RNA splicing, cell differentiation, and metastasis. In the context of the immune system, PRMT7 forms aggregates to catalyze the monomethylation of MAVS at the arginine residue 52 (R52), thereby weakening its binding to TRIM31 and RIG-I. This modification restricts MAVS aggregation and impairs its activation cascade. During viral infection, aggregates of PRMT7 are promptly inactivated through automethylation at arginine residue 32 (R32). Concurrently, MAVS recruits SMURF1 to target PRMT7 for proteasomal degradation, which alleviates PRMT7-mediated suppression and promotes MAVS activation (Zhu et al., 2021a).

### PRMT9

PRMT9 is an uncharacterized type II PRMT that distinguishes itself from other PRMTs through its unique protein sequence. It contains two putative AdoMet-binding motifs and three N-terminal tetratricopeptide repeats (TPR), which contribute to its distinct features (Figure 1B). The precise biological function of PRMT9 still remains elusive. Recent studies have identified PRMT9 as a methyltransferase for several proteins involved in essential biological processes. One of these proteins is SAP145, which plays a role in splicing regulation and has been found to be methylated by PRMT9 (Yang et al., 2015). Additionally, PRMT9 has been implicated in the modulation of the antiviral innate immune response. A recent study reveals that PRMT9 acts as a negative regulator of this immune defense. Overexpression of PRMT9 notably suppresses IFN-β expression and promotes viral replication, whereas PRMT9 knockdown or knockout yields the inverse effects following viral infection. Compared to wild-type mice, those deficient in PRMT9 exhibit reduced susceptibility to RNA virus infections. In an unstimulated state, PRMT9 catalyzes the methylation of MAVS at arginines 41 and 43, a modification that prevents MAVS aggregation and autoactivation, thereby limiting RLR signaling activation. When a viral infection occurs, PRMT9 is dislodged from mitochondria, which leads to a reduction in baseline MAVS methylation levels (Bai et al., 2022). These findings shed light on the complex interplay between PRMT9, protein methylation, and the regulation of immune responses.

## Conclusion and perspectives

Recent advancements have highlighted the potential of PRMTs as potent regulators of the antiviral innate immune response, characterized by the induction of interferon (IFN-I) and interferon-stimulated genes (ISGs) to restrict viral infection. PRMTs can directly interact with innate sensors and downstream adaptors, promoting their methylation. Furthermore, certain PRMTs, such as PRMT6, have been found to regulate antiviral innate immunity beyond their traditional methylation functions. Although arginine methylation has been shown to play a critical role in regulating the antiviral innate immune response, it is worth noting that this is a dynamic modification. Identifying the demethylase or demethylases that counteract the functions of PRMTs in antiviral innate immunity is necessary. In addition to methylation, there have been numerous reports indicating the involvement of other post-translational modifications, such as ubiquitination, phosphorylation, and glycosylation, in the regulation of antiviral responses (Zhou et al., 2017). However, the interactions between these PTMs in the antiviral immune response remain unclear. Therefore, a deeper exploration of the interplay between methylation and other PTMs is crucial. On the other hand, while the majority of research has concentrated on the role of PRMTs in modulating antiviral innate immune signaling, the intricate mechanisms by which viruses influence the regulatory processes of PRMTs in antiviral response remain elusive. Unraveling these mechanisms could unveil new pharmacological targets and treatment strategies for viral infections.

## Author contributions

HN: Data curation, Writing – original draft. QL: Data curation, Resources, Software, Writing – original draft. WP: Conceptualization, Funding acquisition, Supervision, Writing – review & editing.
